# Ingraft chimerism in lung transplantation - a study in a porcine model of obliterative bronchiolitis

**DOI:** 10.1186/1465-9921-12-56

**Published:** 2011-04-26

**Authors:** Outi E Päiväniemi, Petra Musilova, Peter M Raivio, Paula K Maasilta, Hanni S Alho, Jiri Rubes, Kristiina Aittomäki, Ulla-Stina Salminen

**Affiliations:** 1 Department of Cardiothoracic Surgery, Helsinki University Hospital, P.O. Box 340, 00029 HUS, Helsinki, Finland; 2 Department of Genetics and Reproduction, Veterinary Research Institute, Hudcova 70, 621 00 Brno, Czech Republic; 3 Department of Clinical Genetics, Helsinki University Hospital, P.O. Box 63, 00014 University of Helsinki, Finland; 4 Orthopaedic Department, Hatanpää Hospital, P.O. Box 437, 33101 Tampere, Finland

## Abstract

**Background:**

Bronchial epithelium is a target of the alloimmune response in lung transplantation, and intact epithelium may protect allografts from rejection and obliterative bronchiolitis (OB). Herein we study the influence of chimerism on bronchial epithelium and OB development in pigs.

**Methods:**

A total of 54 immunosuppressed and unimmunosuppressed bronchial allografts were serially obtained 2-90 days after transplantation. Histology (H&E) was assessed and the fluorescence in situ hybridization (FISH) method for Y chromosomes using pig-specific DNA-label was used to detect recipient derived cells in graft epithelium and bronchial wall, and donor cell migration to recipient organs. Ingraft chimerism was studied by using male recipients with female donors, whereas donor cell migration to recipient organs was studied using female recipients with male donors.

**Results:**

Early appearance of recipient-derived cells in the airway epithelium appeared predictive of epithelial destruction (*R *= 0.610 - 0.671 and *p *< 0.05) and of obliteration of the bronchial lumen (*R *= 0.698 and *p *< 0.01). All allografts with preserved epithelium showed epithelial chimerism throughout the follow-up. Antirejection medication did not prevent, but delayed the appearance of Y chromosome positive cells in the epithelium (*p *< 0.05), or bronchial wall (*p *< 0.05).

**Conclusions:**

In this study we demonstrate that early appearance of Y chromosomes in the airway epithelium predicts features characteristic of OB. Chimerism occurred in all allografts, including those without features of OB. Therefore we suggest that ingraft chimerism may be a mechanism involved in the repair of alloimmune-mediated tissue injury after transplantation.

## Background

The migration of recipient-derived cells into grafted organs has been demonstrated in heart, liver, and kidney transplants [1-3]. It has been postulated, that these chimeric recipient-derived epithelial and endothelial cells may participate in the graft repair process in lung and liver allografts [2,4]. In lung allografts the bronchial epithelium seems to be a target of the alloimmune response and an intact epithelium is capable of protecting the allografts from chronic rejection i.e. obliterative bronchiolitis (OB). Therefore the integrity of airway epithelium is important for graft preservation [5,6]. Histologically OB is manifested as epithelial cell injury, inflammation, fibrosis, and finally, as obliteration of the small airways [7]. Clinically it is the most important factor limiting long-term survival after lung transplantation [7]. The development of chimerism may also be influenced by antirejection medication [8,9].

After transplantation, donor-derived cells have been detected in the recipient's other tissues, such as lymph nodes, thymus, spleen, and liver [10,11]. The exact role of chimeric cells in transplantation is unclear, but previous studies have proposed that the migration of donor-derived cells into recipient tissues induces tolerance in the recipient [12,13]. To further enhance such systemic chimerism, bone marrow transplantation has been used. In previous studies mixed chimerism has been suggested to increase tolerance in kidney, liver, heart, and lung transplantation [14-18]. In clinical lung transplantation, infusion of donor bone marrow lowered the rate of chronic lung allograft rejection [19]. As the adverse effects of immunosuppressive treatment may also limit the outcome of transplantation, the induction of specific immunological tolerance in the recipient remains an important objective in transplantation.

In this study our goal was to analyse, whether the repaired epithelium in the allograft is recipient-derived, and whether ingraft chimerism in bronchial allografts protects transplants from rejection in a heterotopic porcine model of OB [20]. We also analysed the migration of graft-derived cells into recipient organs.

## Methods

All animals received humane care in compliance with the "Principles of Laboratory Animal Care" formulated by the National Society for Medical Research and the "Guide for the Care and Use of Laboratory Animals" prepared by the Institute of Laboratory Animal Resources and published by the National Institutes of Health (NIH Publication No. 86-23, revised 1996). The study protocol was accepted by the institutional committee for animal research and by the Uusimaa Provincial Administration, Finland (STU828 A). Special attention was given to anesthesia and pain relief during surgical procedures. Animals were euthanized at the end of the study.

### Medication, anesthesia, and surgical procedures

We used 27 random-bred, domestic pigs weighing ca. 20 kg. Animals were anesthetized for the surgical procedures as previously described [21]. Left thoracotomy was performed for removal of the caudal lobe. Peripheral bronchial segments (1-2 cm in length and 1-2 mm in diameter) were dissected free of the surrounding lung parenchyma. These implants were transplanted subcutaneously on the ventral side of each recipient, several implants in all to be removed on each assessment point.

### Study design

To study chimerism in bronchial allografts female animals served as allograft donors while the recipients were male. A male autograft served as a control for the fluorescence in situ hybridization (FISH) method. Allografts without immunosuppressive medication were expected to exhibit rapid destruction of the airway epithelium and obliteration of the lumens of the bronchial allografts. The grafts were followed up to one month. To achieve delayed bronchial obliteration daily oral cyclosporine A 10 mg/kg, methylprednisolone 20 mg, and azathioprine 50 mg were given, and the grafts were followed up to one month. In order to prevent bronchial obliteration recipients received an immunosuppressive regimen of daily oral cyclosporine A 10 mg/kg, methylprednisolone 20 mg, and everolimus 1.5 mg/kg. The total follow-up time of these grafts was three months. At the end of the study, animals were euthanized with a high intravenous dose of sodium pentobarbital.

Bronchial allografts were harvested in the non-treated and inadequately treated groups on days 2, 4, 7, 10, 14, and 28. In the adequately treated group follow-up was extended to days 60 and 90. A total of 54 bronchial samples were fixed in 4% buffered formalin and embedded in wax. Sections 4 μm thick were cut, dewaxed, and stained with hematoxylin and eosin. Epithelial loss and luminal obliteration were graded semi-quantitatively on a scale from 0 (no pathological alteration) to 3 (total epithelial loss, total obliteration).

To further assess chimerism and tolerance induction we studied the migration of donor cells into recipient organs: lung, liver, kidney, and spleen. Five female recipients with male donors were either non-treated, or treated adequately with daily oral cyclosporine A 10 mg/kg, methylprednisolone 20 mg, and everolimus 1.5 mg/kg. In addition, two male recipients with female donors were studied as controls. One was non-treated, and the other one was adequately treated. After a follow-up period of three months, a total of 28 samples were harvested from recipient lung, liver, kidney, and spleen, and prepared similarly to bronchial samples.

### FISH method to detect Y chromosome

The FISH method was used to detect Y chromosomes. Paraffin embedded tissue sections were baked at 56°C for 2-6 h. Deparaffinization was accomplished by immersing the slides in xylene for 3 × 7 min followed by dehydration in ethanol for 2 × 5 min and air drying. Then the slides were sequentially incubated in 0.2 M HCl at room temperature for 25 min, 10 mM citric acid at 80°C for 60 min, 8% sodium thiocyanate at room temperature overnight and 0.025 pepsin/0.01 M HCl at 38°C for 55 min. Each incubation step was followed by washing in 2 × SSC for 2 × 5 min. Finally, the slides were fixed in 4% paraformaldehyde for 11 min, washed in 2 × SSC, dehydrated in ethanol series and air dried. Whole chromosome painting probe specific for pig Y chromosome was prepared by laser microdissection [22]. Satellite probe for centromeric region of chromosome 1, used as hybridization control, was prepared on the basis of Mc1 satellite DNA sequence data (X51555) as described by Rubes et al. [23]. Hybridization mixture (10 μl) containing 50% formamide, 2 × SSC, 10% dextran sulfate, 5 μg salmon sperm DNA, 50 ng of labelled Y chromosome probe and 30 ng labelled chromosome 1 probe was denatured at 80°C for 10 min. Slides were denatured in 70% formamide, 2 × SSC (pH 7.0) at 76°C for 5-10 min. Hybridization was carried out overnight at 37°C. Slides were washed twice in 0.4 × SSC/0.3% igepal (pH 7) at 72°C for 2 min. Fluorescent signals from male recipient epithelium, bronchial wall, and female recipient organs; lung, liver, kidney and spleen, were scored using fluorescent microscopy with magnification 1000× (objective 100× lens and ocular 10×). Findings were graded on a semi-quantitative scale from 0 to 3 based on the number of positive cells (0 = no positive cells, 1 = less than 20% of cells stain positive, 2 = 20% - 50% of cells are positive, 3 = more than 50% cells stain positive).

### Statistics

All data are expressed as mean + SEM. Variations between the groups were calculated by the non-parametric Kruskal-Wallis one-way analysis by ranks. The rank sums were then used for Dunn's test at significance level of 5% (Medstat; Astra Group A/S, Copenhagen, Denmark). Values of *p *< 0.05 were considered significant. For correlation analysis, Spearman's rank correlation (Statistica version 5; StatSoft Inc., Tulsa, OK) was used.

## Results

### Epithelial loss and obliteration

**Figure 1:**
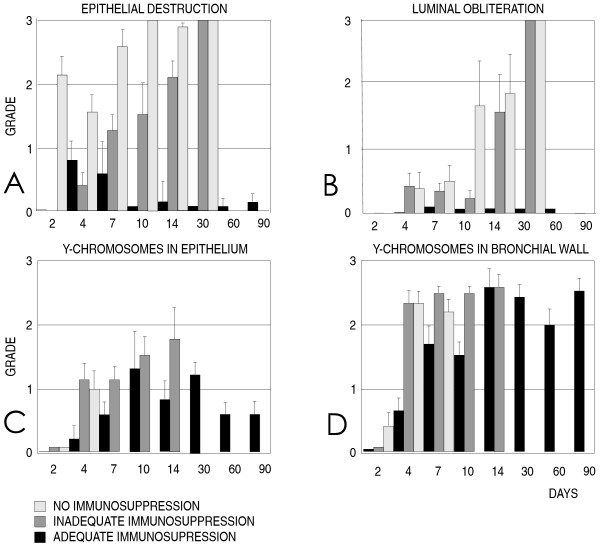
**Destruction of airway epithelium (A) and obliteration of the bronchial lumen (B), and the positive staining for Y chromosomes in the epithelium (C) and in the bronchial wall (D)**. The study groups were non-immunosuppressed, inadequately immunosuppressed, or adequately immunosuppressed. Epithelial destruction, luminal obliteration, and positive staining for Y chromosomes were graded on a scale from 0-3. The number of assessed bronchi in each group and on each assessment point was 6.6. ± 1.1 for histological samples and 7.1. ± 1.1 for FISH.

**Figure 2:**
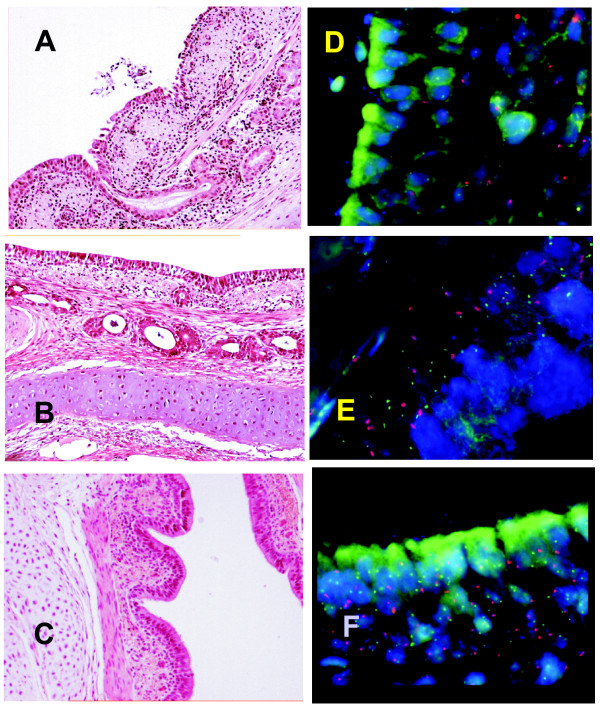
**Table 2. Histological alterations (A,B,C) and chimerism (D,E,F) in bronchial allografts (A,B,D,E) and in a control autograft (C,F)**. Epithelial damage is observed in a bronchial allograft on follow-up day 4 (A) and recovered epithelium and bronchial wall structure on day 7 in an adequately treated allograft (B). *H&E, original magnification ×10*. Recovery of the adequately treated allograft (B) was similar to that of a control autograft (C) on day 7. *H&E, original magnification ×10*. Tissue section of a bronchial allograft of female origin in a male recipient hybridized with probes for pig chromosomes Y (red) and 1 (green) on day 7 (D) shows epithelial and bronchial wall cells with Y chromosomes. Y chromosomes are more frequent on follow-up day 10 (E) than on follow-up day 7 (D) in recovered epithelium in adequately treated allografts. Chromosomes Y (red) and 1 (green) stain invariably (F) in a male control autograft on follow-up day 7 and confirm successful hybridization. *Staining with 4',6-diamidino-2-phenylindole, original magnification ×1000*.

After initial ischemic injury, the respiratory epithelium of allografts with no immunosuppression first showed a tendency to restore. Epithelial loss was subtotal on day 7, and was followed by gradual obliteration of the bronchial lumen (Figures 1A and 1B). In the beginning of the follow-up, the inadequately treated allografts showed less extensive epithelial destruction (Figure 2A), the difference in epithelial loss between the inadequately treated group and non-treated group was significant on follow-up days 4, 7, and 10 (*p *< 0.05). However, by day 30 the lumens of the inadequately treated allografts also occluded totally (Figures 1A and 1B). In the adequately treated group (Figure 2B) and in a control autograft (Figure 2C), only mild epithelial destruction occurred. The difference in epithelial loss between the adequately treated allografts and non-treated allografts was significant from day 7 on (*p *< 0.05). The difference in epithelial loss between the adequately treated allografts and the inadequately treated allografts was significant from day 10 on (*p *< 0.05). No obliterative lesions of the bronchial lumens were observed in adequately treated allografts (Figure 1B). Luminal obliteration of adequately treated allografts differed significantly from non-treated allografts on days 10 and 30 (*p *< 0.05), and from inadequately treated allografts on days 14 and 30 (*p *< 0.05).

### Chimerism

All samples stained positive for chromosome 1 (Figures 2D, E, and 2F) and the control male autograft stained positive for the Y chromosome indicating successful hybridization (Figure 2F). Positive staining for the Y chromosome in bronchial allografts from female donors indicated the presence of recipient-derived cells. Cells showing positive staining for the Y chromosome were observed in all groups both in the respiratory epithelium and in the bronchial wall (Figures 1C and 1D).

Since the respiratory epithelium in the non-treated allografts underwent rapid destruction, epithelial Y chromosomes were observed only at the first assessment points (Figure 1C). In the adequately treated allografts, the number of Y chromosome positive cells in the epithelium increased until day 10 (Figures 1C and 2E). In the respiratory epithelium, Y chromosome positive cells were significantly more numerous on days 10, 14, and 28 than on follow-up day 4 (*p *< 0.05). Positive staining for the Y chromosome was observed in the preserved epithelium during the entire follow-up, up to three months (Figure 1C). Antirejection medication did not prevent, but delayed positive staining for the Y chromosome in the epithelium (*p <*0.05 on day 4) (Figure 1C). Early appearance of Y chromosome positive cells in the airway epithelium appeared predictive of epithelial destruction and obliteration of the bronchial lumen (Table 1).

**Table 1:** Correlations between Y chromosome positivity and features of OB

	Y CHROMOSOMES IN BRONCHIAL EPITHELIUM	
	
	day 4 (N = 21)	day 7 (N = 18)
	
	*R/p*	*R/p*
EPITHELIAL LOSS		
day 10 (N = 21)	0.646/0.004	
day 14 (N = 18)	0.618/0.011	
day 30 (N = 17)	0.610/0.016	

LUMINAL OBLITERATION		
day 14 (N = 18)	0.671/0.024	0.698/0.005

Correlations between epithelial Y chromosome positivity (FISH-method) on follow-up days 4 and 7 and the epithelial loss and luminal obliteration. The bronchial allografts are from female donors and the recipients are male. (N) gives the number of bronchi studied on the assessment points.

Positive staining for the Y chromosome was more numerous in the bronchial wall than in the epithelium on days 4 (*p *= 0.001) and 7 (*p *< 0.0001) and from day 14 onwards until the end of the follow-up (*p *< 0.01) (Figures 1C and 1D). In the bronchial wall, Y chromosome staining was significantly more frequent on follow-up day 14 and thereafter than on day 4 (*p *< 0.05). As in the epithelium, antirejection medication delayed positive staining for the Y chromosome in the bronchial wall (*p *< 0.05, day 4 and 10).

Histologic assessment of the recipient organ samples (lung, liver, kidney and spleen) collected for evaluation of chimerism showed normal histology without adverse effects of medication. The male recipient organs served as controls for the FISH method, and they stained positive for Y chromosomes, whereas organ samples (lung, liver, kidney, and spleen) in female recipients with male bronchial allograft donors were negative. Thus, no donor-derived cells were observed in the recipient organ samples.

## Discussion

In this study we assessed the presence of chimeric cells in bronchial transplants and their relationship to graft rejection. We demonstrated that early appearance of recipient-derived cells in the airway epithelium is predictive of epithelial destruction and obliteration of the bronchial lumen. The non-treated allografts underwent rapid epithelial destruction followed by total luminal obliteration while epithelial loss and subsequent luminal obliteration were slightly delayed in inadequately treated allografts. As expected, bronchial remodelling was prevented in the adequately treated allografts [21], which showed only low-grade epithelial destruction after recovery from the initial ischemic injury. We have previously shown that the advanced epithelial injury occurring in the non-treated or inadequately treated allografts is an alloimmune response with CD4, CD8, and class II positive cell influx [24]. This response results in airway disease with similar histological findings as in human post-transplant OB.

Epithelial chimerism occurred in all groups throughout the follow-up. In the recovering allografts the number of Y chromosome positive cells increased in parallel with the recovery of the respiratory epithelium. Early appearance of recipient-derived cells, however, correlated with further epithelial cell injury and obliteration. It has been shown that epithelial chimerism after transplantation is increased by cellular damage with elevated cell turn over [4,25]. Our finding of low-grade chimerism also in the recovered epithelium of the adequately treated allografts suggests that repair by chimeric cells might be a part of the natural homeostatic mechanism. The adult human lung has a capacity to renew itself by extrapulmonary cells [26] and it has been demonstrated that re-epithelisation with recipient-derived epithelium protects allografts from immune-mediated injury and results in allograft tolerance [27].This was seen also in the present study. The very rapid initiation of chimeric cell migration into the rejected allografts might be an insufficient attempt to protect the epithelium against the alloimmune reaction. The chimeric cells might originate from recipient bone-marrow or hematopoietic stem cells, which are capable of engrafting into the bronchial epithelium of the graft as a part of the repair process [28-30]. On the contrary, whilst the early migration of chimeric cells correlated with subsequent OB, it might be a sign of an active role in this cascade.

An alloimmune injury of the respiratory epithelium is known to result in the secretion of profibrotic factors, which contribute to the development of OB [31]. Murakawa et al found a correlation between the extent of epithelial chimerism and airway remodelling [25]. They also showed that chimeric epithelium prevented luminal fibrosis [25]. In the current study, bronchial wall chimerism occurred in all groups, including allografts without findings of bronchial remodelling. As in the epithelium, medication delayed its occurrence. Recipient-derived myofibroblasts have been suggested to participate in fibrogenesis of the human lung [32]. In addition, chimeric endothelial cells have been detected at sites of endothelial injury [1]. It is unclear, whether recipient-derived endothelial cells are associated with repair or rejection of the graft [1]. The role of bronchial wall chimerism remains unresolved. Similarly to epithelial chimerism, it was present in patent allografts throughout the study and might, thus, be a protection mechanism of the transplanted graft.

Migration of donor-derived cells to recipient tissues after transplantation has been suggested as a mechanism for the initiation and maintenance of tolerance in transplantation. Based on this, donor derived cells would be expected to be present in tissue samples of adequately medicated recipients with preserved allografts [33,34]. In our study, adequately medicated animals did show tolerance against allografts, but we could not detect donor-derived cells in recipient organs, not even after three months follow-up. This finding suggests that tolerance in our model was achieved by immunosuppressive regimen and potential impact of ingraft chimerism, but migration of donor-derived cells into recipient organs did not play a role.

As a limitation of our model and in contrast with clinical lung transplantation, the grafts in our model of OB undergo initial avascularity prior to neovascularisation, and there is a lack of air-epithelial interface. However, the appearance of blood supply is rapid [20]. Animal models are suitable for studies of mechanisms of post-transplant airway disease. However, clinical studies can not be replaced.

## Conclusions

This study shows that early appearance of recipient-derived cells in the airway epithelium is predictive of features observed in post-transplant OB. However, ingraft chimerism might be an attempt to repair tissue damaged as a consequence of the alloimmune response after allograft transplantation. Therefore, methods reinforcing ingraft chimerism might be beneficial after lung transplantation.

## Abbreviations

OB: obliterative bronchiolitis; and FISH: fluorescence in situ hybridization.

## Competing interests

The authors declare that they have no competing interests.

## Authors' contributions

OEP: carried out animal experiments, histology, FISH method and scoring, analysed the results, wrote the paper, performed collaboration with Brno Veterinary Research Institute. PM: carried out FISH method and scoring. PMR: participated in writing. PKM: carried out animal experiments, performed the statistical analysis. HSA: participated in study design and animal experiments. JR: responded to FISH method. KA: performed collaboration with Brno Veterinary Research Institute, FISH design, participated in writing. USS: performed study design, animal experiments, histology and scoring, analysed the results, writing.A ll authors have read and approved the final manuscript.
